# Aberrant Advanced Cognitive and Attention-Related Brain Networks in Parkinson's Disease with Freezing of Gait

**DOI:** 10.1155/2020/8891458

**Published:** 2020-10-08

**Authors:** Yuting Li, Xiuhang Ruan, E. Li, Guoqin Zhang, Yanjun Liu, Yuchen Du, Zhaoxiu Wang, Shaode Yu, Ruimeng Yang, Mengyan Li, Xinhua Wei

**Affiliations:** ^1^Department of Radiology, Guangzhou First People's Hospital, School of Medicine, South China University of Technology, Guangzhou, China; ^2^Department of Radiology, Guangzhou First People's Hospital, Guangzhou Medical University, Guangzhou, China; ^3^Shenzhen Institutes of Advanced Technology, Chinese Academy of Science, Shenzhen, China; ^4^Department of Neurology, Guangzhou First People's Hospital, School of Medicine, South China University of Technology, Guangzhou, China

## Abstract

**Background:**

Freezing of gait (FOG) is a disabling gait disorder influencing patients with Parkinson's disease (PD). Accumulating evidence suggests that FOG is related to the functional alterations within brain networks. We investigated the changes in brain resting-state functional connectivity (FC) in patients with PD with FOG (FOG+) and without FOG (FOG-).

**Methods:**

Resting-state functional magnetic resonance imaging (RS-fMRI) data were collected from 55 PD patients (25 FOG+ and 30 FOG-) and 26 matched healthy controls (HC). Differences in intranetwork connectivity between FOG+, FOG-, and HC individuals were explored using independent component analysis (ICA).

**Results:**

Seven resting-state networks (RSNs) with abnormalities, including motor, executive, and cognitive-related networks, were found in PD patients compared to HC. Compared to FOG- patients, FOG+ patients had increased FC in advanced cognitive and attention-related networks. In addition, the FC values of the auditory network and default mode network were positively correlated with the Gait and Falls Questionnaire (GFQ) and Freezing of Gait Questionnaire (FOGQ) scores in FOG+ patients.

**Conclusions:**

Our findings suggest that the neural basis of PD is associated with impairments of multiple functional networks. Notably, alterations of advanced cognitive and attention-related networks rather than motor networks may be related to the mechanism of FOG.

## 1. Introduction

Freezing of gait (FOG) is a crippling gait characteristic present in Parkinson's disease (PD) patients. PD patients with FOG (FOG+) constantly suffer from falling, leading to a poor quality of life [1, 2]. At present, the treatment of FOG is a very challenging task since the pathogenesis of FOG is not fully understood [2, 3]. The appearance of abnormal gait patterns and rhythm formation disturbances in FOG may be the result of a perceptual malfunction and frontal executive dysfunction [4, 5]. Previous nuclear medicine imaging studies demonstrated that perfusion or metabolism was abnormal in the frontoparietal and the temporal area in FOG+ [6, 7]. Hence, the abnormal function of brain networks may play a considerable role in FOG+.

Some neuroimaging studies repotted that alterations in the functional connectivity (FC) of the locomotor network were responsible for FOG [3, 8]. However, a recent pathophysiological hypothesis suggests that cognitive models, together with decoupling mechanisms, may be the basis of akinetic FOG [9]. A task-based functional magnetic resonance imaging (fMRI) study of PD patients suggested a functional decoupling between movement plan cognition and the inherent motion release in FOG, according to the decoupling model [10]. It should be noted that task-based fMRI increases unpredictability and complexity, which eventually leads to a decline in detection power [11]. Resting-state fMRI (RS-fMRI) is believed to enable the in vivo examination of the patterns of FC on a whole brain scale during rest [12]. Previous RS-fMRI studies have shown that FC within distinct networks and subnetworks in FOG+ patients changes, using voxel-based or seed-based FC analysis [8, 13, 14]. For instance, Lenka et al. [13] performed a seed-to-voxel-based functional analysis with a small sample and suggested that interhemispheric connectivity of the left parietal opercular cortex with the primary somatosensory and auditory areas was reduced in FOG+ patients. Wang et al. [15] set the pedunculopontine nucleus (PPN) as regions of interest (ROIs) to analyze the FC between the local regions and the whole brain, and found that FOG in PD is associated with abnormal corticopontine-cerebellar pathways and the visual temporal areas involved in visual processing.

Many researchers focus on the FC of FOG in PD patients in ROIs, an interesting network or total cerebral FC features. As a functional network connection (FNC) analysis [16], independent component analysis (ICA), however, does not require a priori selection of a seed region and separate the signals of the whole brain into components with statistically independent time courses, resulting in spatially distributed networks without overlap [17]. Recently, a limited body of work explored the alterations of FC in FOG+ using the ICA approach. Tessitore et al. [18] suggested that the disruption of “executive-attention” and visual neural networks was associated with the development of FOG+. Canu et al. [19] revealed poor structural and functional integration between motor and extramotor (cognitive) neural systems in FOG+ patients, and Bharti et al. [20] reported impaired FC in attentive and executive networks in FOG+. However, in the studies of FOG+ with ICA methods, the sample size was relatively small in two studies: one study was carried out on an MR scanner under 3.0 T in a magnetic field, and one study did not include FOG- as a comparison group. Moreover, the medication status of the patients was not consistent in these studies.

In the current study, we performed RS-fMRI to investigate the alterations in FC within resting-state networks (RSNs) using the ICA approach, and to reveal the correlation between the abnormal brain network and clinical features in FOG+ patients in the ON state. We hypothesized that multiple functional networks would be altered in FOG+ patients, and that changes in cognitive and executive attention-related brain networks rather than motor networks would play a primary role in the development of FOG.

## 2. Materials and Methods

### 2.1. Participants and Clinical Assessments

A total of 56 PD patients from the Parkinson's and Movement Disorders Clinic of the Guangzhou First People's Hospital, including 31 FOG- and 25 FOG+ patients, and 26 healthy controls (HC) from community recruitment were enrolled in the study. The demographic and clinical features of the subjects are summarized in Table 1. The diagnosis of PD was made according to the clinical criteria of the Movement Disorder Society [21] by a senior PD specialist in neurology with 25 years of working experience. The criteria for the exclusion of PD patients were as follows: (i) secondary Parkinsonism; (ii) a history of mental illnesses; (iii) a history of surgical operations; (iv) cognitive dysfunction (Mini Mental State Examination (MMSE) [22] score < 24); and (v) prohibition from MRI scanning procedures, such as due to having metal embedded in the body. Patients were classified as FOG+ based on the following two conditions: (i) a score > 0 on item 4 (evaluating whether FOG is present) of the Gait and Falls Questionnaire (GFQ, score/64) and a score > 0 on items other than 1 and 2 of the Freezing of Gait Questionnaire (FOGQ score/24) [23]; (ii) in addition to the description of FOG by patients, FOG could be verified by the senior PD specialist. The patients who did not meet the above conditions were FOG-. PD patients were also clinically assessed with other scales, including the Hoehn & Yahr scale (H&Y) [24] to evaluate the severity of PD symptoms, the Unified Parkinson's Disease Rating Scale (UPDRS), the PDQ-39 [25]—a short 39-item quality of life questionnaire for PD—and the MMSE. HC with no history of neuropsychiatric diseases, no symptoms of PD, and no history of surgical operations was recruited for the assessment of PD and FOG-related effects in relation to the normal population.

The study protocol was approved by the Clinical Research Ethics Committee of Guangzhou First People's Hospital, Guangdong Province, China. Written informed consent was provided by each participant in accordance with the Declaration of Helsinki (2008 version).

### 2.2. Imaging Parameters

All subjects were scanned in a 3.0 T Verio MRI scanner (Siemens, Erlangen, Germany) equipped with an 8-channel parallel head coil and were required to lie quietly in the scanner while staying awake with their eyes closed. All of the PD patients were in a medication-on state during MRI inspection. Both functional and structural images were obtained. The resting-state functional images were acquired with echo-planar imaging (EPI) with the following parameters: repetition time (TR) = 2000 ms; echo time (TE) = 21 ms; slice thickness/gap = 4 mm/0.6 mm; acquisition matrix = 64 × 64; flip angle = 78°; voxel size = 3.5 mm × 3.5 mm × 4.0 mm; and field of view (FOV) = 224 × 224 mm^2^. Sagittal T1-weighted images were obtained with the following parameters: TR/TE = 1900 ms/2.19 ms; acquisition matrix = 256 × 256; flip angle = 9°; voxel size = 1.0 mm × 1.0 mm × 1.0 mm; slice thickness/gap = 1 mm/0.5 mm.

### 2.3. Data Preprocessing

Implemented on the MATLAB R2013a platform, functional images were preprocessed using DPABI (version 3.0 http://rfmri.org/dpabi) software, the RS-fMRI Data Analysis Toolkit (REST) (version 1.8 http://restfmri.net/forum/REST_V1.8), and Statistical Parametric Mapping (SPM 8 https://www.fil.ion.ucl.ac.uk/spm/software/spm8/). Data preprocessing included the following steps: (i) convert DICOM into NIFTI; (ii) remove the first 10 of the 220 time points in case of unstable signal quality; (iii) perform slice-timing adjustment (30 slices); (iv) perform realignment, excluding subjects with maximal head motion exceeding 2 mm or rotations over 2 degrees; (v) conduct spatial normalization to the EPI template of Montreal Neurological Institute (MNI) space by resampling to 3 mm × 3 mm × 3 mm; (vi) remove linear detrending; (vii) smooth at 8 mm full width at half maximum (FWHM); and (viii) perform regression of nuisance covariates (including white matter, cerebrospinal fluid, and head motion). One FOG- patient was excluded during realignment.

### 2.4. Group Independent Component Analysis

Functional images were obtained with spatial group independent component analysis in a data-driven manner, via the GIFT version 3.0b toolbox (http://mialab.mrn.org/software/gift). As a very general-purpose statistical technique, ICA identifies random data that are linearly transformed into components that are maximally independent from each other in reliable temporal relationships [26]. To ensure sufficient decomposition and appropriate splitting of the major networks, 30 independent components (ICs) were extracted with the Infomax algorithm. The ICs with differences were matched with eight RSN templates provided by Dante Mantini from KU Leuven Medical School [27], including ventral attention network (VAN), auditory network (AUN), default mode network (DMN), dorsal attention network (DAN), bilateral frontoparietal network (LFPN/RFPN), somatomotor network (SMN), and visual network (VIN). Since the VAN could not identify the three groups, 7 of 8 statistically meaningful RSNs were identified as anatomically and functionally classical RSNs.

One-sample *t*-tests were performed on *z* score spatial maps across all participants in SPM 8 to determine regions positively significantly integrated into each component at a voxel-level family-wise error- (FWE-) corrected *p*_FWE_ < 0.01 combined with a cluster extent threshold of 20 voxels, following the combination of the three masks as the statistical range of the analysis of variance (ANOVA). The combined mask was used in the *post hoc* analysis of functional connectivity differences between every two groups by ANOVA with age and sex as covariates. The ROIs showing significant brain connectivity differences were visualized using the xjview 9.7 toolbox (https://www.alivelearn.net/xjview).

### 2.5. Statistical and Correlative Analysis

Statistically significant differences among the three groups in terms of demographic and clinical data were performed by a Pearson chi-square test, ANOVA, a Kruskal-Wallis H-Test, and a Mann-Whitney *U* test, as appropriate. Relationships between ROIs, extracted from ICA and clinical assessments, including the GFQ, FOGQ, and PDQ-39, were explored with correlations. The above statistical analyses were performed in SPSS version 25.0 software (https://developer.ibm.com/predictiveanalytics/downloads), and the level of statistical significance was set at *p* < 0.05.

## 3. Results

### 3.1. Demographic Characteristics and Clinical Assessments

Ultimately, 30 FOG-, 25 FOG+, and 26 HC individuals were included after realignment. The demographic characteristics and clinical assessments of both PD patients and HC are summarized in Table 1. Importantly, FOG+ patients were older than FOG- and HC participants (*p* = 0.001), while no significant difference was found between FOG- and HC participants (*p* > 0.05). Compared to FOG- participants, FOG+ participants had longer disease durations and more serious PD symptoms (H&Y, UPDRS-II, and UPDRS-IV), lower quality of life (PDQ-39), and higher GFQ and FOGQ scores. However, FOG+ and FOG- participants demonstrated no significant differences (*p* > 0.05) on MMSE, UPDRS-I, and UPDRS-III scores.

### 3.2. Group Independent Component Analysis and Correlative Analysis

No significant difference in the altered FC among FOG+, FOG-, and HC participants was found in the VAN; however, the remaining 7 RSNs, including the AUN, DMN, DAN, FPN (LFPN/RFPN), SMN, and VIN, exhibited statistically meaningful regional differences in their distributions (Table 2). More details of the brain regions of the RSNs could be found in the supplementary material (available here).

**Figure 1:**
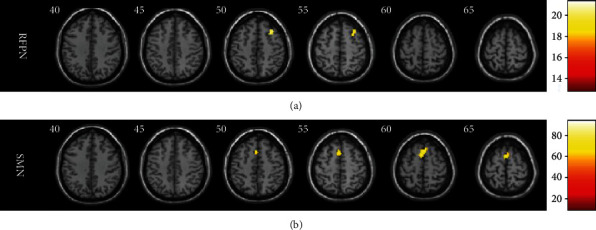
Aberrant functional connectivity in the RFPN and the SMN among the three groups. (a, b) Statistical maps for the RFPN and the SMN among the three groups. RFPN: right frontoparietal network; SMN: somatomotor network.

Figure 1 indicates that there was no significant difference in the functional changes between FOG+ and FOG- in the RFPN and the SMN. Compared with HC, however, the whole group of PD patients showed higher FC in the SMN, and only FOG- participants exhibited higher FC in the RFPN.

**Figure 2:**
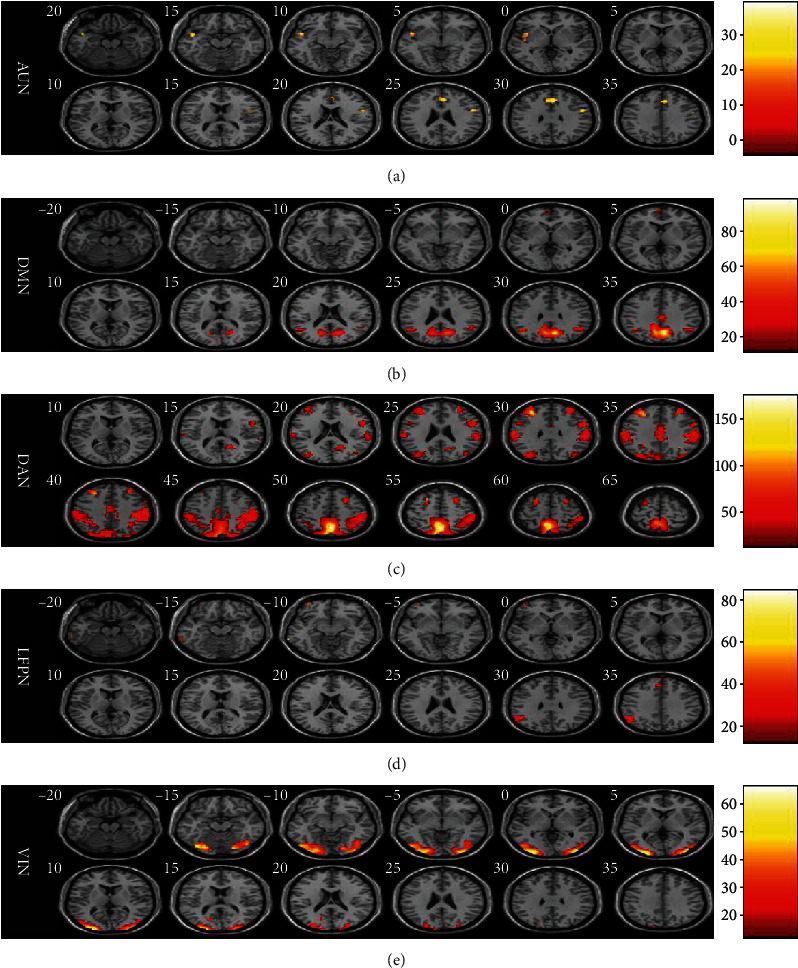
Aberrant functional connectivity in the AUN, DMN, DAN, LFPN, and VIN among the three groups. (a–e) Statistical maps for the AUN, DMN, DAN, LFPN, and VIN among the three groups. AUN: auditory network; DMN: default mode network; DAN: dorsal attention network; LFPN: left frontoparietal network; RFPN: right frontoparietal network; VIN: visual network.

**Figure 3:**
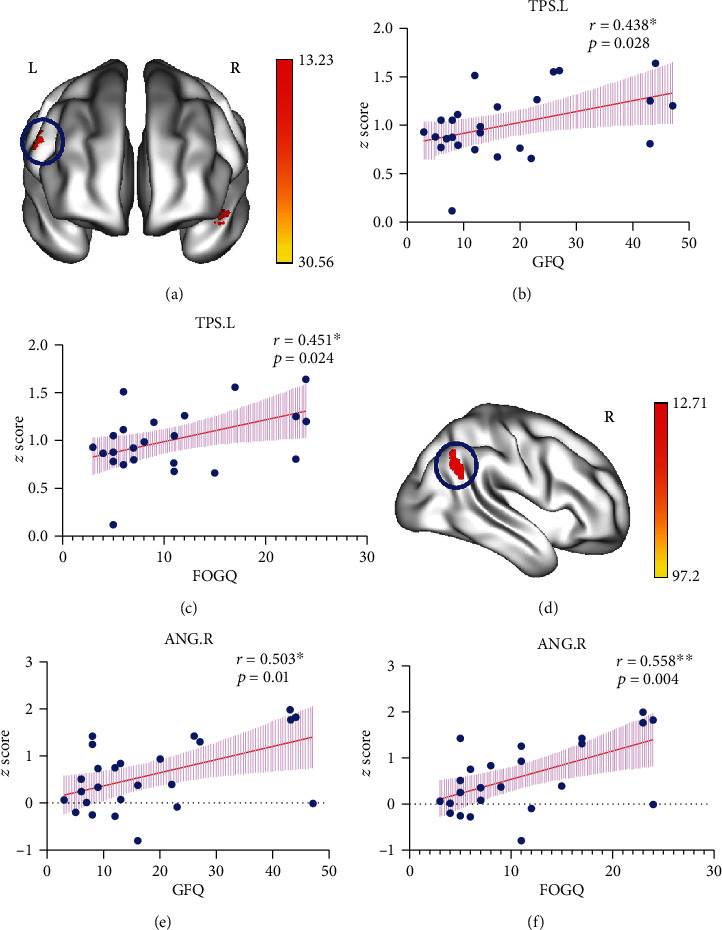
The correlations between brain regions connectivity abnormities and the severity of gait disorders symptoms in FOG+ patients. (a) Threshold maps for the TPS.L of the AUN. (b, c) Altered connectivity in the TPS.L correlated positively with GFQ score (*p* = 0.028, rho = 0.438) and FOGQ score (*p* = 0.024, rho = 0.451). (d) Threshold maps for the ANG.R of the DMN. (e, f) Altered connectivity in the ANG.R correlated positively with GFQ score (*p* = 0.01, rho = 0.503) and FOGQ score (*p* = 0.004, rho = 0.558). FOG+: Parkinson's disease with freezing of gait; L/R: left/right hemisphere; AUN: auditory network; TPS: temporal pole: superior temporal gyrus; DMN: default mode network; ANG: angular gyrus; GFQ: Gait and Falls Questionnaire; FOGQ: Freezing of Gait Questionnaire.

In the AUN, DMN, DAN, LFPN, and VIN, significant differences were found among the three groups (Figure 2). In particular, FOG+ participants displayed increased FC in TPS.L (left temporal pole: superior temporal gyrus) of the AUN compared with that of the other groups, which was positively correlated with the GFQ score (*p* = 0.028; rho = 0.438) and FOGQ score (*p* = 0.024; rho = 0.451) (Figures 3(a)–3(c)). Moreover, we observed lower FC in the ANG.R (right angular gyrus) of the DMN, which was positively correlated with the GFQ score (*p* = 0.01; rho = 0.503) and FOGQ score (*p* = 0.004; rho = 0.558) (Figures 3(d)–3(f)).

## 4. Discussion

Based on the ICA method, we performed RS-fMRI research to investigate the alterations of FC within the whole brain networks in PD patients with and without FOG during the ON state. Our results reveal that FC was significantly changed in 5 RSNs, including the AUN, DMN, DAN, LFPN, and VIN, which are advanced cognitive and attention-related areas, in FOG+ patients compared with FOG- patients. Moreover, the FC of the AUN and DMN was positively correlated with the GFQ and FOGQ scores in FOG+ patients. Also, we found that the whole group of PD patients showed altered FC in the AUN, DMN, DAN, LFPN, RFPN, SMN, and VIN, compared with HC.

### 4.1. Abnormal Functional Connections between FOG+ and FOG

Our studies illustrated that brain network differences in FC between FOG+ and FOG-patients were within the AUN, DMN, DAN, LFPN, and VIN, which are advanced cognitive and attention-related regions. In fact, we observed that focused attention in life can overcome FOG; however, using cognitive load to divide attention would increase the occurrence of FOG [18]. Different from the previous researches explored the alterations of FC in FOG+ using the ICA approach, we found that the lessening of FC in the AUN was positively correlated with GFQ and FOGQ scores supported the finding that abnormal connections in the AUN are indeed the cause of FOG. Hearing impairment may be one of the reasons why PD patients often suffer from gait disorders such as falls because perception and action complement each other [28]. We found increased FC in the PCUN.L and ANG.L of the DMN in FOG+ patients, but Canu et al. [19] found decreased FC in the DMN, which might be the effect of dopaminergic medication because Zhong et al. [29] found that levodopa has the ability to intensify DMN connectivity in PD patients in the ON state. Interestingly, we observed FC in the ANG.R of the DMN decreased in FOG+ patients, which was positively related to GFQ and FOGQ scores. A previous study suggested that the gray matter of the inferior parietal lobe (IPL), including the ANG, atrophied in FOG+ patients [30]. The IPL participates in the sensory integration of perceptual spatiotemporal information, and the functional defects of the IPL may lead to a disrupted control of and a bilateral incoordination of gait, which can explain why FOG+ patients suffer from brief and sudden episodic inability to take a step despite the intention to walk [30, 31]. A study found that the dorsal attention pathway rather than the ventral attention pathway plays a leading role in FOG [32], which is consistent with our results. The DAN manages spatial attention and visual movement and regulates the top-down guided voluntary allocation of attention, which plays an important role in the implementation of cognitive strategies required for gait [33, 34]. The lessening of FC in the DAN indicated visual spatial attention deficit and thus leads to FOG. Working memory could reflect cognitive function [35]. We observed that FC in the LFPN increased in FOG+ patients. Therefore, we inferred that in FOG+ patients, the LFPN, which is related to working memory, showed compensatory hyperactivation to maintain behavior in brain network deficits [36]. At the same time, we observed that the FC in the bilateral middle occipital gyrus within the VIN was reduced in FOG+ patients compared that in FOG- patients, which is partly consistent with those reported by Tessitore et al. [18], who observed reduced FC in the right occipitotemporal gyrus of the VIN. Visual defects are associated with gait disorders and greater disabilities [37]. Visual dependence may compensate for motor impairment in FOG+ patients and thus visual cues contribute to the improvement of gait [38]. Overall, FOG is associated with brain network abnormalities related to advanced cognition and attention, including auditory, visual, and working memory defects, the DMN, and visual spatial networks.

However, the functional connections located in the RFPN and the SMN, which are related to execution and motion, respectively, were not significantly different between FOG+ and FOG- patients. Tessitore et al. [18] found that FC in the RFPN decreased in FOG+ patients, even though these patients usually exhibit impairments in executive attention function even during the earliest stages of the disease, while Bharti et al. [20] observed increased FC in the RFPN in FOG+ patients. It has been shown that the executive attention function of PD patients is affected differently by dopaminergic medication, and most of them benefitted from the treatment [39]. Hence, we consider that long-term drug therapy may play a compensatory role in FOG+ patients compared with its potential role in FOG- patients with a relatively shorter drug therapy course. A growing body of imaging studies has shown that the FC of the motor area is altered in FOG+ [8, 13]. Nevertheless, we should note that a lack of coordination in patients exists not only in the legs but also in the arms [40]. FOG patients have greater variability in determining which swinging limb to use to initiate gait than FOG- individuals, suggesting that response selection disorders (or cognitive impairment) may interfere with coupling at movement initiation [41]. Therefore, the abnormal preaction during gait initiation may show difficulties during conflict resolution or may even indicate the failure of the motor program through the “alternative network” while trying to overcome obstacles [9].

### 4.2. Abnormal Functional Connections between HC and PD Patients with and without FOG

In addition to the changes in the above brain networks in FOG+ patients, compared with HC, FOG+, and FOG- patients share common functional alterations within the AUN, DMN, DAN, LFPN, SMN, and VIN. In addition, the FOG- group showed lower FC in the RFPN than the HC group. Our results indicate the pathophysiology of cognitive, executive attention, and motor dysfunction in patients with PD, which is in line with a previous review [42]. It is worth noting that the somatomotor FC of the whole group of PD patients was altered, but there was no significant difference between FOG+ and FOG- patients, which indicates that abnormal motor function is common in PD but not a specific manifestation of FOG.

## 5. Limitations

Some important limitations should be taken into consideration when interpreting our results. First, there is a lack of functional connection analysis between networks. The generation, processing, and transmission of brain information require cooperation between networks [12]. Second, the demographic characteristics of age were not properly matched among the three groups. The age of FOG+ patients was significantly higher than that of the other groups. We observed that PD patients who were older and had longer disease durations were more likely to suffer from FOG [43]. In recent research, age was regressed as a covariable in statistical analysis to eliminate mismatched confounding factors. In the further research, we would include data of older HC and older FOG- patients to exclude the possibility that age affected the results-differences in FC between FOG+ and FOG- patients. Third, several studies used the ICA method to analyze the aberration in PD patients with FOG before, but we have inconsistencies in sample size, subject grouping, medication status, and results. Finally, the aim of this kind of gait, movement disorders examined by RS-fMRI involves some errors, maybe during movement, FC will change. More research is necessary to detect the changes of the structural networks that explain FOG.

## 6. Conclusion

The present study shows that PD is associated with abnormal cerebral functional activity in multi-RSNs and that FOG is a result of decoupling between action cognition and its initiation. Based on these findings, we believe that advanced cognitive and attention-related brain networks may play a more important role than motor networks in the neural mechanism of FOG. Despite some limitations, we provide a possible neural mechanism for understanding FOG, which is of particular significance for clinical intervention in PD patients with FOG.

## Figures and Tables

**Table 1:** Demographic characteristics and clinical assessments.

	HC (*n* = 26) mean (SD)	FOG- (*n* = 30) mean (SD)	FOG+ (*n* = 25) mean (SD)	*p* value
Age (yrs)	60.19 (3.783)	60.00 (10.498)	66.52 (8.574)	0.001^a^
Male/female	11/15	17/13	15/10	0.397^b^
Disease duration (yrs)	NA	2.72 (2.98)	6.86 (5.37)	<0.001^c^
H&Y	NA	2.03 (0.41)	2.60 (0.69)	0.002^c^
MMSE	27.58 (2.06)	27.97 (1.83)	27.12 (1.80)	0.204^a^
UPDRS-I	NA	1.43 (1.65)	2.00 (2.20)	0.382^c^
UPDRS-II	NA	6.60 (3.04)	12.32 (8.11)	0.002^d^
UPDRS-III	NA	25.10 (13.79)	29.16 (18.42)	0.368^d^
UPDRS-IV	NA	0.97 (1.90)	3.20 (2.99)	0.001^c^
PDQ-39	NA	16.43 (11.93)	34.20 (26.66)	0.006^c^
GFO	NA	2.83 (2.45)	17.84 (13.47)	<0.001^c^
FOGQ	NA	1.50 (1.46)	10.72 (6.89)	<0.001^c^

HC: healthy controls; FOG+/FOG-: Parkinson's disease with/without freezing of gait; NA: not applicable. H&Y: Hoehn & Yahr; MMSE: Mini Mental State Examination; UPDRS: Unified Parkinson's Disease Rating Scale; GFQ: Gait and Falls Questionnaire; FOGQ: Freezing of Gait Questionnaire. ^a^Statistical *p* value was obtained by Kruskal-Wallis *H*-test. ^b^Statistical *p* value was obtained by Pearson Chi-Square test. ^c^Statistical *p* value was obtained by Mann-Whitney *U* test. ^d^Statistical *p* value was obtained by Independent Student *t-*test.

**Table 2:** Brain regions in resting-state networks (RSNs) with significant differences in functional connectivity among FOG+, FOG-, and HC participants.

RSNs/regions (AAL)	Cluster size (mm^3^)	Peak MNI coordinates (*x* *y* *z*)			Peak *T*-value
		*X*	*Y*	*Z*	
AUN					
TPS.L	104	-45	3	-15	24.1194
PreCG.R	45	54	3	27	30.5567
MCG.R	96	6	30	33	29.8302
DMN					
SFGmed.L	21	-3	66	6	30.5884
PCUN.L	1297	6	-63	36	97.2039
ANG.L	95	-48	-57	27	42.8589
ANG.R	109	51	-54	36	28.296
DAN					
ITG.R	56	51	-60	-9	23.913
PCUN.R	64	15	-57	15	45.5962
IFGoperc.R	182	57	9	27	62.9567
SMG.R	1117	42	-27	42	69.4657
PCUN.L	2853	3	-63	54	175.8931
LFPN					
ITG.L	20	-66	-48	-12	84.2057
ORBmid.L	20	-33	57	-12	41.5616
RFPN					
MFG.R	27	33	21	51	21.3332
SMN					
SMA.L	152	3	3	72	24.0919
VIN					
MOG.R	631	39	-90	3	65.4637
MOG.L	817	-27	-99	9	66.4165

The *T*-value was obtained by post hoc analysis of one-sample *t*-tests, corrected *p*_FWE_ < 0.01, cluster extent threshold of 20 voxels. FOG+/FOG-: Parkinson's disease with/without freezing of gait; HC: healthy controls; MNI: Montreal Neurological Institute; L/R: left/right hemisphere; AUN: auditory network; TPS: temporal pole: superior temporal gyrus; PreCG: precentral gyrus; MCG: median cingulate and paracingulate gyrus; DMN: default mode network; SFGmed: superior frontal gyrus, medial; PCUN: precuneus; ANG: angular gyrus; DAN: dorsal attention network; ITG: inferior temporal gyrus; IFGoperc: inferior frontal gyrus, opercular part; SMG: supramarginal gyrus; LFPN: left frontoparietal network; ORBmid: middle frontal gyrus, orbital part; RFPN: right frontoparietal network; MFG: middle frontal gyrus; SMN: somatomotor network; SMA: supplementary motor area; VIN: visual network; MOG: middle occipital gyrus.

## Data Availability

The data of this study are available on reasonable request from the corresponding author.

## Supplementary Materials

Supplementary Materials Supplementary Table: comparison of FC alterations in RSNs among FOG+, FOG-, and HC participants.
