# Supplementary material for: Aberrant Advanced Cognitive and Attention-Related Brain Networks in Parkinson's Disease with Freezing of Gait
Source: Neural Plast. 2020 Oct 8;2020:8891458. doi: 10.1155/2020/8891458 (PMC7568140; doi:10.1155/2020/8891458)
Supplement: Supplementary Materials — Supplementary Table: comparison of FC alterations in RSNs among FOG+, FOG-, and HC participants. [file 8891458.f1.docx]

Supplementary table: Comparison of FC alterations in RSNs among FOG+, FOG- and HC participants.

| Group | RSNs | Regions |
| --- | --- | --- |
| *FOG+ vs FOG-* |  |  |
| FOG+ > FOG- | AUN | TPS.L |
|  | DMN | PCUN.L, ANG.L |
|  | DAN | IFGoperc.R |
|  | LFPN | ITG.L, ORBmid.L |
| FOG+ < FOG- | AUN | PreCG.R, MCG.R |
|  | DMN | ANG.R |
|  | DAN | ITG.R, PCUN.R, SMG.R, PCUN.L |
|  | VIN | MOG.R, MOG.L |
| *FOG+ vs HC* |  |  |
| FOG+ > HC | AUN | TPS.L |
|  | DMN | SFGmed.L, PCUN.L, ANG.L |
|  | DAN | ITG.R, SMG.R, PCUN.L |
|  | LFPN | ITG.L |
|  | SMN | SMA.L |
|  | VIN | MOG.R, MOG.L |
| FOG+ < HC | AUN | PreCG.R, MCG.R |
|  | DMN | ANG.R |
|  | DAN | PCUN.R, IFGoperc.R |
|  | LFPN | ORBmid.L |
| *FOG- vs HC* |  |  |
| FOG- > HC | DMN | SFGmed.L, PCUN.L, ANG.L |
|  | DAN | ITG.R, SMG.R, PCUN.L |
|  | SMN | SMA.L |
|  | VIN | MOG.R, MOG.L |
| FOG- < HC | AUN | PreCG.R, MCG.R |
|  | DMN | ANG.R |
|  | DAN | PCUN.R, IFGoperc.R |
|  | LFPN | ITG.L, ORBmid.L |
|  | RFPN | MFG.R |

PD > HC represents higher FC in the whole group of PD patients than healthy controls (HC). PD_FOG > FOG- represents higher FC in PD patients with FOG (FOG+) than in PD patients without FOG (FOG-). PD < HC represents lower FC in the whole group of PD patients than HC. FOG- < HC represents lower FC in FOG- than in HC. FOG+< FOG- represents lower FC in FOG+ than in FOG-. The results were statistically obtained by *post hoc* analysis of one-sample *t*-test, corrected *p*_FWE_<0.01, cluster extent threshold of 20 voxels. FOG+/FOG-, Parkinson’s disease with/without freezing of gait; HC, healthy controls; MNI, Montreal Neurological Institute; L/R, left/right hemisphere; AUN, auditory network; TPS, Temporal pole: superior temporal gyrus; PreCG, Precentral gyrus; MCG, Median cingulate and paracingulate gyrus; DMN, default mode network; SFGmed, Superior frontal gyrus, medial; PCUN, Precuneus; ANG, Angular gyrus; DAN, dorsal attention network; ITG, Inferior temporal gyrus; IFGoperc, Inferior frontal gyrus, opercular part; SMG, Supramarginal gyrus; LFPN, left frontoparietal network; ORBmid, Middle frontal gyrus, orbital part; RFPN, right frontoparietal network; MFG, Middle frontal gyrus; SMN, somatomotor network; SMA, Supplementary motor area; VIN, visual network; MOG, Middle occipital gyrus.
